# Metagenomic Identification of Novel Eukaryotic Viruses with Small DNA Genomes in Pheasants

**DOI:** 10.3390/ani14020237

**Published:** 2024-01-12

**Authors:** Eszter Kaszab, Krisztina Bali, Szilvia Marton, Krisztina Ursu, Szilvia L. Farkas, Enikő Fehér, Marianna Domán, Vito Martella, Krisztián Bányai

**Affiliations:** 1 HUN-REN Veterinary Medical Research Institute, 1143 Budapest, Hungary; kaszab.eszter@vmri.hun-ren.hu (E.K.); bali.krisztina@vmri.hun-ren.hu (K.B.); marton.szilvia@vmri.hun-ren.hu (S.M.); feher.eniko@vmri.hun-ren.hu (E.F.); doman.marianna@vmri.hun-ren.hu (M.D.); 2 National Laboratory for Infectious Animal Diseases, Antimicrobial Resistance, Veterinary Public Health and Food Chain Safety, 1143 Budapest, Hungary; 3 One Health Institute, Faculty of Health Sciences, University of Debrecen, 4032 Debrecen, Hungary; 4 Veterinary Diagnostic Directorate, National Food Chain Safety Office, 1143 Budapest, Hungary; ursuk@nebih.gov.hu; 5 Department of Obstetrics and Food Animal Medicine Clinic, University of Veterinary Medicine, 1078 Budapest, Hungary; farkas.szilvia@univet.hu; 6 Department of Veterinary Medicine, University of Bari Aldo Moro, 70010 Valenzano, Italy; vito.martella@uniba.it; 7 Department of Pharmacology and Toxicology, University of Veterinary Medicine, 1078 Budapest, Hungary

**Keywords:** virome, genome assembly, phylogenetic analysis

## Abstract

**Simple Summary:**

In this study, we report the diversity of viruses identified using metagenomics in the intestinal contents of dead pheasants. The study provides evidence for the presence of a heterogenous viral community, with an over-representation of viruses with small DNA genomes. This information will be useful for the development of diagnostic methods that can be used in routine health assessment and virus surveillance.

**Abstract:**

A panel of intestinal samples collected from common pheasants (*Phasianus colchicus*) between 2008 and 2017 was used for metagenomic investigation using an unbiased enrichment protocol and different bioinformatic pipelines. The number of sequence reads in the metagenomic analysis ranged from 1,419,265 to 17,507,704 with a viral sequence read rate ranging from 0.01% to 59%. When considering the sequence reads of eukaryotic viruses, RNA and DNA viruses were identified in the samples, including but not limited to coronaviruses, reoviruses, parvoviruses, and CRESS DNA viruses (i.e., circular Rep-encoding single-stranded DNA viruses). Partial or nearly complete genome sequences were reconstructed of at least three different parvoviruses (dependoparvovirus, aveparvovirus and chaphamaparvovirus), as well as gyroviruses and diverse CRESS DNA viruses. Generating information of virus diversity will serve as a basis for developing specific diagnostic tools and for structured epidemiological investigations, useful to assess the impact of these novel viruses on animal health.

## 1. Introduction

Common pheasants (*Phasianus colchicus*) are among the most popular game bird species, and millions of pheasants are reared across the world for sporting purposes. Game birds are usually farmed semi-intensively at a younger age and then extensively before being released to the wild [1,2]. Game bird species are well-known reservoirs of pathogenic agents for chickens and turkeys, as they belong to the same avian taxon, Galliformes. Viral diseases affecting intensively housed poultry (e.g., Newcastle disease, infectious bronchitis, influenza A virus, parvovirus infections, etc.) are also able to cause considerable losses for game bird breeders [1,2,3]. 

The literature on the viral etiology of enteric disease syndromes in these species is still incomplete. In pheasants, viral intestinal problems have been linked to rotaviruses and caliciviruses [4]. However, viral enteric diseases (e.g., poultry enteritis and mortality syndrome or PEMS) in other galliform birds have been shown to be multi-etiological health problems [5], suggesting that similar syndromes in game birds can be more complex than currently recognized. Metagenomic characterization of viruses from pheasants with intestinal disease is an obvious option to help elucidate the viral spectrum associated with gut infection in this avian species. The timely identification of pathogenic viruses infecting juvenile pheasants seems to be crucial for the implementation of adequate preventive measures during the breeding and rearing period. 

In this study, we report the results of viral metagenomics conducted on the intestinal content of pheasants and extend current knowledge on the complexity of pheasant viral flora.

## 2. Materials and Methods

### 2.1. Samples

Remainders of pooled gut samples (*n* = 20) were processed for viral metagenomic analysis. Originally, the pheasant carcasses were submitted for routine pathology and virology testing between 2008 to 2017 from various pheasant farms located in five Hungarian counties. Available background information linked to the samples is given in Table 1. Intestines were removed and archived at −80 °C after diagnostic processing in the respective detection years. The primary diagnosis based on pathology examination was typically PEMS-like disease and virology tests [6,7,8,9] showed positivity for avian rotaviruses (*n* = 12), avian reoviruses (*n* = 5), coronaviruses (*n* = 3), astroviruses (*n* = 2) and/or adenoviruses (*n* = 2). Additionally, *E. coli* and Coccidia were also detected in some samples.

### 2.2. Sample Processing

Metagenomics-related laboratory methods used in our laboratory have been described elsewhere in detail [10]. In brief, the thawed intestines were homogenized in sterile phosphate-buffered saline and then centrifuged at 10,000× *g* for 5 min at room temperature. The supernatants were filtered using a 0.45 µm PES filter (Nantong FilterBio Membrane Co., Ltd., Nantong, China). The viral nucleic acids were extracted using a NucleoSpin RNA Virus Kit (Macherey-Nagel, Düren, Germany) according to the manufacturer’s instruction. After sequence-independent amplification using random primed RT-PCR, the amplicons were cleaned up with a Gel/PCR DNA Fragments Extraction Kit (Geneaid Biotech Ltd., Taipei, Taiwan). Libraries for next-generation sequencing were prepared with a Nextera XT DNA Sample Preparation Kit (Illumina, San Diego, CA, USA) and subjected to sequencing on the Illumina NextSeq 500 platform according to a previously described protocol [10].

### 2.3. Data Processing

For bioinformatics analysis, single-end reads of ~150 nucleotides (nt) were generated. The FASTQ datasets were analyzed with (viral) metagenomic pipelines and classification tools: Centrifuge v1.0.4. (database h + p + v) [11], Kraken2 v1.0 (database PlusPF) [12], Kaiju (webserver, database NCBI BLAST nr +euk) [13], and Virmine v2.0 [14]. One of the four pipelines’ assembled sequence reads de novo (Virmine), whereas the others classified unassembled reads. All these pipelines are publicly available as open-source software, but Kaiju also has a web-based option [13]. For the remainder of analyses, to visualize differences in the virus community composition, we summarized the data of output files manually and displayed the higher taxonomic levels (e.g., genera, families) or relevant viruses on the plots. To confirm our results, mapping of raw reads was performed against reference genomes of the viruses of interest using Geneious Prime 2022.2.2 (https://www.geneious.com) (Biomatters, Auckland, New Zealand). The full or partial consensus sequences of the viruses were queried against the database of BLASTN (https://blast.ncbi.nlm.nih.gov/Blast.cgi) to identify virus sequences. 

Nearly complete sequences for some viruses were manually edited by using Geneious Prime. The obtained complete genomes were annotated with the Geneious Prime 2022.2.2. Additional open reading frames (ORFs) were predicted with the ORF Finder tool (https://www.ncbi.nlm.nih.gov/orffinder/). For phylogenetic analysis, we built datasets for all viruses detected in the study. Sequence alignments were created with the MAFFT plugin of Geneious Prime. Maximum-likelihood trees were inferred with MEGA X or PhyML 3.0 using the best model for each data set and applying 500 bootstrap replicates [15,16]. Sequence identity values were calculated using the Geneious Prime software.

## 3. Results

### 3.1. Viral Flora of the Pheasant Intestine

A total of 20 pools of pheasant intestinal specimens were analyzed using viral metagenomics. Samples were collected from a period encompassing 10 years. In some years, several pools were tested in parallel from the same locations (Table 1). In general, 89,199,122 reads were generated for these 20 samples using next-generation sequencing in a range of 1,419,265 to 17,507,704 sequence reads for each sample; of these, the specimen with the largest sequence data was available from a previous report and was reanalyzed in this study [17]. Sequence reads were analyzed using four metagenomic classifiers: Kraken, Kaiju, Centrifuge, and Virmine. However, an initial evaluation of the results from our dataset obtained by Virmine showed very high rate of false positives for virus hits; therefore, the Virmine results were excluded from the evaluation.

**Figure 1:**
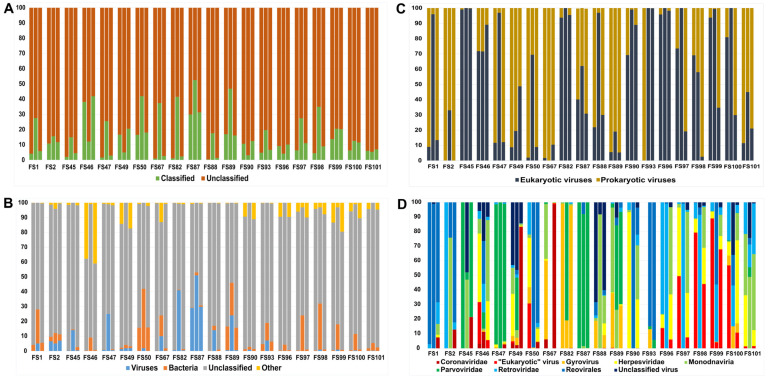
Results of the NGS experiments on the enteric samples, expressed as % values, as decoded using Kraken (left column), Kaiju (central column) and Centrifuge (right column). (**A**) Ratio of classified vs. unclassified reads; (**B**) composition of the reads based on viral, bacterial, or ‘other’ origin; unclassified reads are also displayed; (**C**) ratio of eukaryotic vs. prokaryotic reads; (**D**) composition of viral reads based on taxonomical attribution.

Sequence classifications were evaluated in four consecutive steps. (i) First, we sorted sequence reads based on whether they were classified taxa or represented unclassifiable sequences. The Kaiju pipeline yielded the highest whereas Kraken gave the lowest rates of classified sequence reads. The range of classified reads was 0.4% to 38.1% using Kraken, 3.2% to 52.6% using Kaiju and 1.4% to 41.9% using Centrifuge (Figure 1A). (ii) Then, we focused on the ratio of viral reads. Among the three methods, Kaiju resulted in the greatest rates of virus hits (per sample range, 0.02% to 51%), followed by Kraken and Centrifuge with comparable per-sample virus sequence reads (Kraken, <0.01% to 59%, Centrifuge, 0.02% to 29.8%) (Figure 1B). (iii) When comparing the reads of prokaryotic viruses (i.e., bacteriophages) and eukaryotic viruses (i.e., viruses of eukaryote hosts), we observed marked differences among samples. The rate between bacteriophages and eukaryotic viruses also varied among the pipelines. Prokaryotic viruses dominated in four samples, eukaryote viruses in eight samples. Very high rates of prokaryote viruses (≥90%) were identified in eight samples as predicted by at least one of the three classifiers, whereas comparable high rates of eukaryote viruses (>90%) were detected in seven samples (Figure 1C). (iv) Finally, we collated the classification of different eukaryotic viruses. Viruses of eukaryotic hosts included both DNA viruses (such as gyroviruses, herpesviruses, ‘monodnaviruses’, and parvoviruses) and RNA viruses (coronaviruses, reoviruses and rotaviruses), including RNA viruses with DNA stage (retroviruses). Other eukaryotic viruses and unclassified viruses were also identified; among these were some hits that corresponded to the properties of some small circular DNA viruses (including chicken stool-associated circular virus 1 and -2, sewage-associated circular DNA virus-28) (Figure 1D). 

**Figure 2:**
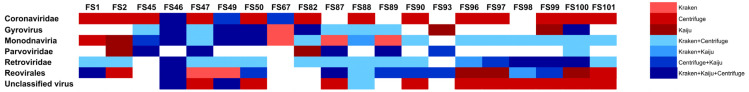
Combined detection of different viruses in the pheasant intestinal content by using three different metagenomic classifiers (Monodnaviria here does not include parvoviruses and gyroviruses).

Importantly, all 20 sample pools were found to contain eukaryotic viruses (Figure 2). Moreover, when results from the three bioinformatic classifiers were combined, three eukaryotic viral taxa were detected in two samples and, in a similar way, four, five, six and seven viruses were predicted in two, seven, six and two sample pools, respectively. Coronaviruses, gyroviruses, monodnaviruses (all viruses with circular DNA genome except for gyroviruses), parvoviruses, retroviruses, and reoviruses (orthoreovirus and rotavirus, combined) were detected in 80%, 65%, 100%, 40%, 85%, and 85%, respectively, of samples. Of interest, several samples that showed positivity for rotaviruses (nine out of twelve) or coronaviruses (two out of three) using diagnostic RT-PCR also yielded sequence reads using metagenomics. 

### 3.2. Characterization of Novel Viruses

When assembling the short sequence reads to generate longer contigs by de novo assembly, or mapping to reference sequences assigned as best hits, herpesvirus, retrovirus and coronavirus assemblies yielded low-quality contigs, typically no larger than a few hundred base pairs in length. These viruses are not further discussed in this study. Additionally, given that the identified representatives of *Reovirales* did not constitute a group of novel virus species (only rotaviruses and orthoreoviruses were detected), they are not discussed in the present study either.

The following section will focus on three major groups of eukaryotic viruses with the smallest single-stranded DNA genomes. These include several viruses possibly representing new taxa.

#### 3.2.1. Novel Parvoviruses

We identified representative members of three parvovirus genera in the pheasant intestinal content, such as dependoparvoviruses in three samples, aveparvovirus in one sample and chaphamaparvoviruses in three samples.

**Figure 3:**
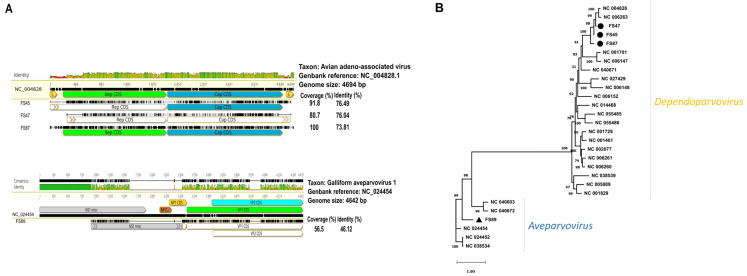
(**A**) Comparative genomic illustration of the identified dependoparvovirus sequences with their reference sequences. (**B**) Maximum likelihood phylogenetic tree of representative capsid nt sequences of *Dependoparvovirus* and *Aveparvovirus* genus (PhyML 3.0, TNR93 + R model, aLRTSH-like branch support). Branch support values lower than 80 were hidden. Sequences identified in this study are highlighted with dots.

As for FS87, an avian adeno-associated parvovirus (dependoparvovirus) genome was assembled from 769,904 reads that represented 49.8% of all reads generated for this sample. The average sequencing depth was 19,238× for the full-length genome. The genome was 4717 nt in length and encoded Rep (2001 nt, 667 aa) and Cp (2244 nt, 748 aa) and shared 74% nt identity with the reference genome (NC_004828, Avian adeno-associated virus ATCC VR-865, Figure 3). Concerning FS47, only a partial genome could be assembled; in particular, a 617 nt-long fragment was missing from the 5′ end of the genome affecting the rep gene whose coverage reached roughly 80% of the reference. Another 61 nt was missing from the 3′ end of the cap gene plus the entire non-coding region downstream of the cap gene. The 2253 reads (~0.1% of the 2,314,548 reads generated for this sample) allowed the assembly of 56.6% of the predicted genome. Similar contig size was determined for FS45, with missing 307 nt from the 5′ end, with the full-length cap gene (in this case, 2238 bp, 746 aa) and largely undetermined downstream region after the cap region. The obtained genome was assembled at a 2252× depth from 9844 sequence reads. The capsid protein coding gene, cap, showed some genetic variation among sequences with inter-strain identity values of 78.7% to 93%. The three Hungarian pheasant dependoparvoviruses shared high nt sequence identities to each other (range: 94.1–96.4%).

Concerning the single aveparvovirus sequence (found in sample pool FS89), roughly half of the genome was assembled from 18,145 sequence reads (i.e., 1.2% of all reads). Gaps came from missing sequence reads located at the beginning and in the middle of the predicted genome structure. The genome with all gaps was most similar to a turkey parvovirus strain, 1078 (NC_024454), a finding that was confirmed using phylogenetic analysis (Figure 3). The concatenated genomic sequence shared 65% identity to the reference turkey origin aveparvovirus strain, which was found to be the most closely related viral taxon.

**Figure 4:**
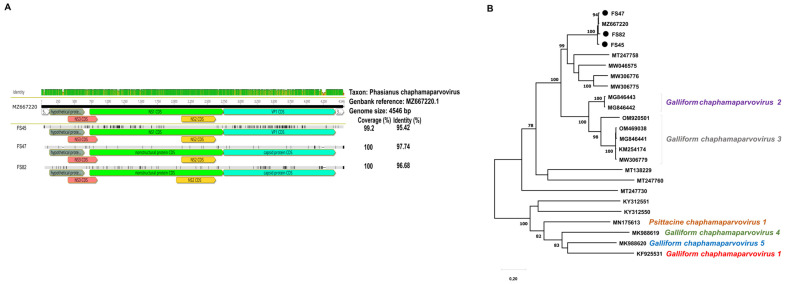
(**A**) Comparative genomic illustration of the identified chaphamaparvovirus sequences with the reference sequence. (**B**) Maximum likelihood phylogenetic tree of representative NS1 aa sequences of *Chaphamaparvovirus* genus (MEGA-X software, LG+G+I+F pmodel, 500 bootsrap replicates). Branch support values lower than 70 were hidden. Sequences identified in this study are highlighted with dots.

The genomes of three chaphamaparvoviruses were almost completely determined. These genomes were assembled from a large portion of raw reads (FS45, 8.1%; FS47, 81.6%; FS82, 60.5%), resulting in considerable sequence depths for the three genomes (ranging from 4750× to 71,384×). These results showed that pheasant chaphamaparvoviruses constituted a major component of the metagenomic assemblage in the tested sample pools. The genome lengths varied as follows: FS45, 4510 nt; FS47, 4454 nt; FS82, 4458 nt. The NS1 and NS3 genes showed no variation in length among genomes (NS1, 2022 nt; NS3, 444 nt), whereas the VP1 (1653 nt to 1689 nt), NS2 (519 nt to 597 nt) and hypothetical protein coding ORF (504 to 525 nt) showed sequence length differences among samples. The three Hungarian strains shared 94.1 to 96.4% genome sequence nt identity and 95.4 to 97.7% to the reference pheasant chaphamaparvovirus strain from France (Figure 4). The second and third most closely related chaphamaparvoviruses, strain bls219par05 from black swan (*Cygnus atratus*) and strain CTCPaV1/CT08.18-AU-2018 from chestnut teal (*Anas castanea*), shared, respectively, up to 68.8% and 69.6% genome-wide nt identity to pheasant-origin chaphamaparvoviruses.

#### 3.2.2. Novel Gyroviruses

**Figure 5:**
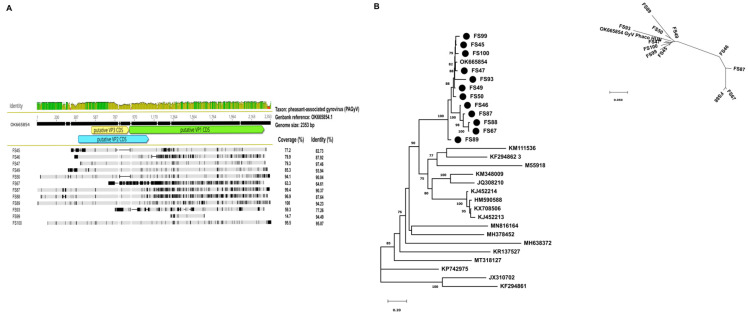
(**A**) Comparative genomic illustration of the identified gyrovirus sequences with the reference sequence. (**B**) Maximum likelihood phylogenetic tree of representative partial VP1 nt sequences of *Gyrovirus* genus (PhyML 3.0, HKY85+G+I model, aLRTSH-like branch support). Branch support values lower than 70 were hidden. Sequences identified in this study are highlighted with dots.

To our surprise, a total of 12 sample pools were found to contain traits of gyroviruses. Interestingly, different classifiers showed different abilities to identify gyroviruses; Kaiju identified three gyroviruses and Kraken found gyrovirus in one sample, whereas Kraken plus Centrifuge and the three methods combined together found gyrovirus sequences in five and four sample pools, respectively. Two complete genomes were assembled. FS93 and FS100 contained the largest ratio of gyrovirus-specific reads (13% and 15%, respectively), whilst in other samples we identified as low as 0.03–0.6% gyrovirus-specific sequence reads. Complete or nearly complete genomes were assembled from samples containing 246 to 851 virus-specific reads with a sequencing depth ranging from 11× to 40×. In the remainders, with lower sequence quality, partial genome sequences (spanning 14.6 to 85.2% of the predicted genome) were obtained at sequencing depths ranging from <1× to 2523×. The four strains, whose full-length genomes were reconstructed, shared 88 to 95.9% identity at the genomic level (Figure 5).

The three ORFs (ORF1, ORF2 and ORF3) were predicted to code for proteins of VP1 (with a length of 455 aa or 456 aa), VP2 (235 aa) and VP3 (126 aa), respectively. The VP1 coding region is used in species demarcation. The full-length VP1 region displayed 91.8 to 97.1% nt and 90.5 to 98% aa identities among each other.

#### 3.2.3. Novel CRESS DNA Viruses

The initial metagenomic assessment identified 19 sample pools containing CRESS DNA viruses (circular Rep-encoding single-stranded DNA) or other unusual members of the realm *Monodnaviria*, but the majority of these hits were not confirmed in subsequent analyses. As a result, the genomic traits of three distantly related groups of CRESS DNA viruses were identified in five sample pools. These three viral groups were classified as chicken-stool-associated circular viruses, genomoviruses and proprismacoviruses. For simplicity, the chicken-stool-associated circular viruses, which are currently unclassified viruses within the realm *Monodnaviria*, are also listed here.

**Figure 6:**
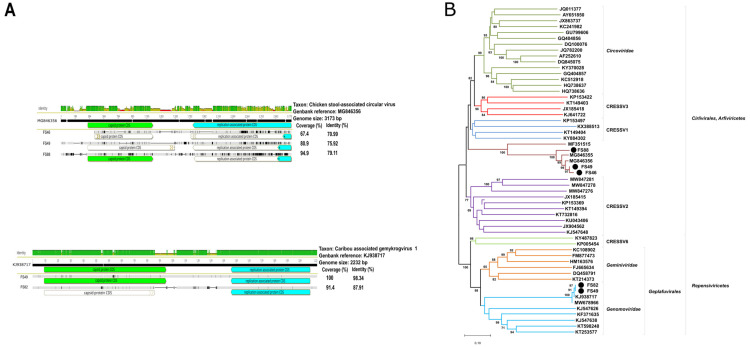
(**A**) Comparative genomic illustration of the selected CRESS DNA sequences with their reference sequences. (**B**) Neighbor-joining phylogenetic tree of representative partial Rep aa sequences of CRESS DNA viruses (MEGA-X software, bootstrap tests of 1000 replicates, p-distance model). Branch support values lower than 60 were hidden. Sequences identified in this study are highlighted with dots.

Genomic sequences related to the chicken-stool-associated circular virus were found in three samples: FS88, FS46 and FS49. However, full-length genomes could not be assembled from the shotgun sequencing reads. The best quality sequence was obtained for FS88. In this sample, a full-length *cp* gene (909 bp, 303 aa) and a poorly assembled *rep* gene constituted the hypothetical genome sequence and only 351 of the 1,820,438 sequence reads assembled into this contig. Multiple gaps in the *rep*, *cp* and non-coding regions interrupted the genome of the two other samples, FS46 and FS49, even if the number of sequence reads used was higher (FS46, 1707 reads; FS49, 2315 reads). The concatenated contigs gave a genome-wide coverage for the three partial genomes, 67.4% to 94.9%, and highest similarity to reference sequence with range of 71 to 79%. Taking the sequence qualities into account, the phylogenetic tree was prepared using an aa sequence alignment that contained sequences of 206–448 residues of the Rep (Figure 6).

Concerning genomoviruses, the sequence reads from FS82 and FS49 assembled into a 2040 nt and a 2232 nt contig by reference mapping, respectively (Figure 6). Concerning FS82, the genomovirus-specific contig was assembled from 1004 reads (out of 17,507,704 total reads, at a sequencing depth of 52×), whereas the FS49 was assembled from 128 reads (out of 1,470,963 total reads at a depth of 7×).

**Figure 7:**
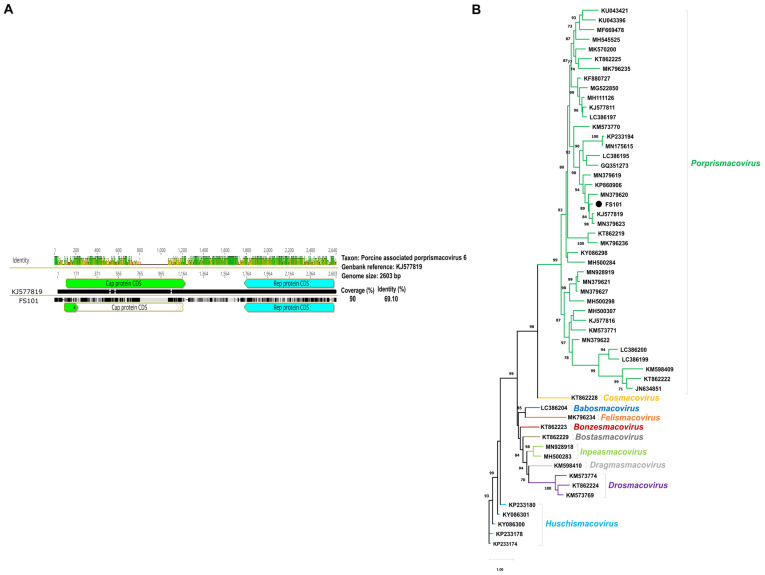
(**A**). Comparative genomic illustration of the identified proprismacovirus sequence with the reference sequence. (**B**). Maximum likelihood phylogenetic tree of representative *rep* aa sequences of *Smacoviridae* (PhyML 3.0, Q.fam+G+I+F model, aLRTSH-like branch support). Branch support values lower than 70 were hidden. Sequences identified in this study are highlighted with dots.

Proprismacovirus was detected in a single sample pool. A total of 6272 reads (out of 4,474,049, 0.1%) mapped to a GenBank reference strain XP1 (NC_024776) and were assembled into a 2328 nt long fragment of the putative genome. This was roughly 90% of the expected genome size and included the full-length *rep* gene and nearly full-length *cp* gene. The *rep* gene shared 75% and 78.9% identity at the nt and aa level, respectively, with the most closely related reference strain, XP1. The expected genome-wide similarity at this 90% coverage was approximately 69.1% (Figure 7).

## 4. Discussion

Viral metagenomics faces several challenges that prevent it from being included in the repertoire of routine diagnostic methods. A major challenge is that, unlike bacteria and eukaryotic organisms, viruses do not share a common genetic marker that would serve as shared target for the development and application of universal amplification methods with broad reactivity. Other problems include the different capsid and nucleic acid stability across virus families and the varying efficacies of virus enrichment protocols that are difficult to adapt and standardize in diagnostic laboratories. Moreover, available bioinformatics pipelines show various efficacies for detection of viral sequences in samples of interest. Thus, viral metagenomics has limited use in diagnostic laboratories but remains a promising discovery tool of viral diversity in biological or environmental samples [18].

In this study, we analyzed archived samples whose majority (85%) was stored >10 years before being processed. No samples from the most recent years were available. By using metagenomic tools, viral reads were found in various proportions in the pooled samples, ranging from <0.01% to 59%. This is consistent with findings of other reports showing that low percentages of viral reads in metagenomic assemblages are a typical finding [19,20,21]. Despite these shortcomings, both RNA and DNA viruses were detected with our protocols. However, it is of note that virus-specific PCR results and metagenomics data did not fully match. For example, astrovirus-specific sequence reads were not generated at all, although some samples tested positive using standard PCR assays. Similarly, detection rates of other RNA viruses were lower with metagenomics than expected from routine laboratory results obtained with specific PCR assays. It is very likely that these discrepancies were at least partly related to either low virus titers of the samples or depletion of viral RNA under storage conditions.

Two distantly related groups of DNA viruses (within the realm *Monodnaviria*) prevailed in the samples, since parvoviruses were found in 25% of samples and gyroviruses in 60%. Although prevalence rates cannot be calculated as the samples were pooled before laboratory procedures, these data show the frequent occurrence of some DNA viruses in the pheasant intestine.

Parvoviruses are small, icosahedral, non-enveloped, single-stranded DNA viruses of approximately 4 to 6 kilobase in length [22,23,24]. The genome of parvoviruses typically contains two major coding regions encoding non-structural (NS) proteins and structural proteins (VPs). Parvoviruses use alternative splicing to form one or more non-structural proteins (NS1-NS3), in which the replication initiator protein NS1 plays the main role in the life cycle of virus. With alternative splicing, up to four VPs (VP1-4) can be generated from the ORF of VP. With metagenomic approaches, numerous animal parvoviruses have been discovered in recent years, challenging the taxonomy of the family *Parvoviridae*. Currently, viruses within the family *Parvoviridae* are divided into three subfamilies: *Parvovirinae* (10 genera), *Densovirinae* (11 genera) and *Hamaparvovirinae* (5 genera) [22,23,24].

In recent years, members of the genus *Chaphamaparvovirus* (subfamily *Hamaparvovirinae*) have been discovered in the feces or in various tissue samples of avian (e.g., chickens, turkeys, peafowl, cranes, parakeets, canaries, owl, etc.) and mammalian (rats, pigs, dogs, cats, bats, etc.) reservoirs [22,24,25,26]. Viruses similar to the chaphamaparvovirus detected in this study have been identified in France in different tissue samples of pheasants with hepatitis [27]. Also, chaphamaparoviruses have been associated with enteritis in cats [28] and hedgehogs [29], with nephropathy and kidney fibrosis in mice [30] and with systemic disease in bearded dragons [31], suggesting a potential pathogenic role of these parvoviruses in divergent vertebrate hosts. Thus, the etiological role of these viruses in avian species surely deserves further studies.

Dependoparvoviruses obtained their name based on their dependency on the simultaneous cellular infection with another virus. Most members of this taxon are adenovirus-associated and require helper viruses to complete their viral infection cycle [32]. However, some avian dependoparvoviruses are autonomous and replicate in tissues of growing birds [32]. Partial or nearly complete genome sequences of three dependoviruses were generated in this study from the archival pheasant samples. The viruses appeared to be genetically related to each other (94.1–96.4% nt identity), hinting to a possible species-specific pattern. Of interest, two sample pools that were positive for dependoparvovirus by metagenomics were also positive for adenovirus by virus-specific PCR (FS87 and FS88, Table 1).

A third parvovirus type, classified as aveparvovirus, was also identified in our metagenomic investigations. Based on partial genome sequence, the virus appeared to be distantly related to turkey aveparvovirus. Aveparvoviruses have been described in several countries in chicken and turkey and are mostly associated with enteric disease [32]. Genetically related aveparvoviruses have been identified in pigeons [33] and in pileated finches [34].

Gyroviruses belong to the *Anelloviridae* family. The gyrovirus genome is circular ssDNA and measures 2.2 to 2.6 kilobase. Gyroviruses occur in mammals and birds; in mammals, the source of detection is typically the feces, while in birds, gyroviruses can also be detected in tissue samples [35]. With the exception of chicken anemia virus (*Gyrovirus chickenanemia*), which is an immunosuppressive agent in chicken, other gyrovirus species are not known to cause diseases in the avian host species in which they have been identified, or the putative pathologic findings have yet to be confirmed [36,37,38,39,40,41,42,43]. In this study, we identified gyrovirus genomic traits in 12 sample pools originating from 5 counties between 2008 and 2011. Genetic variability was demonstrated among sequences with a range of sequence identity of 92% to 97% when analyzing the VP1 gene. This level of sequence divergence may suggest that pheasants serve as true host species for the identified gyrovirus species, tentatively called *Gyrovirus phaco 1* [17].

CRESS DNA viruses comprise a rapidly growing group of viruses. The diversity of these viruses seems to be unparalleled in the kingdom of viruses. They possess small ssDNA circular genomes (e.g., smacoviruses, 2.3–3 kb; genomoviruses, 2.2–2.4 kb). Many host species are known to carry CRESS DNA viruses and identical or very closely related CRESS DNA viruses are found occasionally in distant host species, suggesting a relatively broad host range for selected virus species [44,45]. Both wild birds and domestic poultry are considered to be hosts for numerous CRESS DNA viruses [46,47]. However, none of the CRESS DNA viruses identified in this study or their distantly related heterologous counterparts have been linked to any diseases of avian hosts. More interestingly, recent evidence questions whether eukaryotic organisms are the true hosts for some of these viruses [48,49,50].

## 5. Conclusions

The viral flora of common pheasants is poorly studied using laboratory tools of metagenomics [18]. Pheasants are widely reared game birds that are released into the wild when they reach adulthood. Samples for this study were collected during the rearing phase while birds were kept in captivity. It would be interesting to collect additional data on released or hunted pheasants to see how their viral flora changes over time in relation to exposure to new environmental conditions. The genome sequences of novel DNA viruses described here could guide future studies to design specific diagnostic tests. Such tests could be utilized to investigate the potential role of identified viruses in the observed pathologies and to initiate virus epidemiological surveys to gain information regarding the transmissibility and host range of the identified viral agents.

## Figures and Tables

**Table 1:** Sample characteristics, including year and location of sampling, pathologies and diagnostic findings.

Year	Location (County)	Sample	Clinical Signs/Pathology	Laboratory Findings *
2008	Komárom-Esztergom	FS2	acute enteritis, weak chicks, bone formation disorder	RV, *E. coli*
	Fejér	FS93	premature, dehydrated bird with acute enteritis	RV, *E. coli*
	Somogy	FS96 to FS100	enteritis	ReoV
2010	Somogy	FS1	enteritis	RV
2011	Somogy	FS45	enteritis	RV, *E. coli*, coccidiosis
	Fejér	FS46	enteritis	RV, coccidiosis
		FS49	enteritis	RV
	Komárom-Esztergom	FS87	emaciated, dehydrated bird with enteritis	AstV, AdV
		FS88	emaciated, dehydrated bird with enteritis	AstV, AdV, CoV, *E. coli*
		FS89	emaciated, dehydrated bird with enteritis	CoV
		FS90	emaciated, dehydrated bird with enteritis	RV, CoV, *E. coli*
2012	Bács-Kiskun	FS47	enteritis	RV, *E. coli*
2013	Győr-Moson-Sopron	FS50	desquamative enteritis, serous hepatits, tubulonephrosis	RV
2014	Bács-Kiskun	FS101	enteritis	RV, *E. coli*
2016	Fejér	FS67	uneven growth, enteritis, tubulonephrosis, liver degeneration, mortality	RV, *E. coli*
2017	Bács-Kiskun	FS82	enteritis	RV

* Abbreviations: RV, rotavirus; ReoV, reovirus; AstV, astrovirus; AdV, adenovirus; CoV, coronavirus; *E. coli*, *Escherichia coli*.

## Data Availability

Raw sequences have been deposited in the SRA database (accession no., PRJNA1032468).
